# Certain dietary patterns are associated with GLIM criteria among Chinese community-dwelling older adults: a cross-sectional analysis

**DOI:** 10.1017/jns.2021.64

**Published:** 2021-08-27

**Authors:** Suey S. Y. Yeung, Ruth S. M. Chan, Jenny S. W. Lee, Jean Woo

**Affiliations:** 1 Department of Medicine and Therapeutics, Faculty of Medicine, The Chinese University of Hong Kong, Hong Kong, China; 2 Department of Applied Biology and Chemical Technology, The Hong Kong Polytechnic University, Hong Kong, China; 3 Department of Medicine, Alice Ho Miu Ling Nethersole Hospital, Hong Kong, China; 4 Department of Medicine and Geriatrics, Tai Po Hospital, Hong Kong, China; 5 Centre for Nutritional Studies, Faculty of Medicine, The Chinese University of Hong Kong, Hong Kong, China

**Keywords:** Aged, Chinese, GLIM criteria, Dietary patterns, Diet quality, Malnutrition, BMI, body mass index, CI, confidence intervals, CSID, Community Screening Instrument for Dementia, DASH, Dietary Approaches to Stop Hypertension, DQI-I, Dietary Quality Index International, hsCRP, high-sensitivity C-reactive protein, GLIM, Global Leadership Initiative on Malnutrition, MDS, Mediterranean Diet Score, OR, odds ratio, PASE, Physical Activity Scale for the Elderly

## Abstract

Disease-related malnutrition is prevalent among older adults; therefore, identifying the modifiable risk factors in the diet is essential for the prevention and management of disease-related malnutrition. The present study examined the cross-sectional association between dietary patterns and malnutrition in Chinese community-dwelling older adults aged ≥65 years in Hong Kong. Dietary patterns, including Diet Quality Index International (DQI-I), Dietary Approaches to Stop Hypertension (DASH), the Mediterranean Diet Score, ‘vegetable–fruit’ pattern, ‘snack–drink–milk product’ pattern and ‘meat–fish’ pattern, were estimated and generated from a validated food frequency questionnaire. Malnutrition was classified according to the modified Global Leadership Initiative on Malnutrition (GLIM) criteria based on two phenotypic components (low body mass index and reduced muscle mass) and one aetiologic component (inflammation/disease burden). The association between the tertile or level of adherence of each dietary pattern and modified GLIM criteria was analysed using adjusted binary logistic regression models. Data of 3694 participants were available (49 % men). Malnutrition was present in 397 participants (10⋅7 %). In men, a higher DQI-I score, a higher ‘vegetable–fruit’ pattern score and a lower ‘meat–fish’ pattern score were associated with a lower risk of malnutrition. In women, higher adherence to the DASH diet was associated with a lower risk of malnutrition. After the Bonferroni correction, the association remained statistically significant only in men for the DQI-I score. To conclude, a higher DQI-I score was associated with a lower risk of malnutrition in Chinese older men. Nutritional strategies for the prevention and management of malnutrition could potentially be targeted on dietary quality.

## Introduction

Malnutrition has been defined as ‘a state resulting from lack of uptake or intake of nutrition causing altered body composition, leading to diminished physical and mental function and impaired outcome from disease’. Malnutrition can result from starvation, disease or advanced aging, alone or in combination^(1)^. Since multimorbidity is prevalent among older adults^(2)^, exploring disease-related malnutrition in this population is highly relevant. Disease-related malnutrition is associated with morbidity, sarcopenia, loss of independence, hospitalisation and mortality in older adults^(3–5)^. Consumption of a healthy diet reduces the risk of developing chronic diseases and malnutrition^(6)^. Identifying the modifiable risk factors in the diet is therefore essential for the prevention and management of malnutrition.

Diets are composed of food and nutrients; therefore, focusing on isolated food groups or nutrients cannot account for the synergistic and/or antagonistic interactions between nutrients^(7)^. In this context, a dietary pattern approach to examine the role of diet on malnutrition is preferred over an individual food group or single nutrient approach. Previous studies have shown inconsistent findings regarding the association between diet quality and malnutrition in community-dwelling older adults^(8–11)^. Two prospective studies found that diet quality was not associated with malnutrition defined by weight status^(8)^ and the Mini-Nutritional Assessment (MNA)^(9)^. In contrast, two cross-sectional studies found that adherence to the Mediterranean diet and high-nutrient-dense dietary pattern was associated with a lower risk of malnutrition assessed with the Determine Your Nutritional Health Checklist^(10)^ and weight status^(11)^ respectively. Recently, a consensus scheme for diagnosing malnutrition has been proposed by the Global Leadership Initiative on Malnutrition (GLIM)^(12)^. Since dietary pattern is a potentially modifiable risk factor for malnutrition, studies of the association between dietary patterns and malnutrition according to the GLIM criteria are therefore of value.

Given the scarcity of evidence on this topic and the fact that dietary culture varies among countries, the present study examined the association between dietary patterns and disease-related malnutrition according to GLIM criteria among Chinese community-dwelling older adults in Hong Kong. The hypothesis for the present study was that healthier dietary patterns are associated with a lower risk of malnutrition according to the GLIM criteria.

## Methods and materials

### Study population

This was a cross-sectional analysis of a prospective cohort study (Mr. and Ms. Os study). In the years 2001–2003, 2000 men and 2000 women aged 65 years and above living in the community in Hong Kong were invited to attend a health check conducted at the University Teaching Hospital in Shatin^(13)^. Recruitment notices were placed at the common areas in local elderly community centres and housing estates. Participants were volunteers and should be able to travel to the study site. Those who were unable to walk without the assistance of another person, had a bilateral hip replacement or were not competent to give informed consent were excluded. The target was to recruit a stratified sample that equally assigned them into each of these age groups: 65–69, 70–74 and ≥75. The study was conducted according to the guidelines laid down in the Declaration of Helsinki, and all procedures involving human subjects were approved by the Clinical Research Ethics Committee of the Chinese University of Hong Kong (CRE: 2003.102). Written informed consent was obtained from all participants.

In the present study, we excluded 298 participants with incomplete data to classify malnutrition according to the GLIM criteria. We further excluded those from the analysis due to extremely high (>5000 kcal) or low (<500 kcal) energy intake^(14–16)^. The final sample size for the present analysis was 3694.

### Participants’ demographics

A structured interview was conducted to collect information on age, sex, smoking habit, alcohol use, living status, marital status, education level, subjective social status and medical history. Subjective social status was assessed using a 10-rung self-anchoring scale. Participants were asked to mark their self-perceived position on an upright ladder with 10 rungs. The lowest rung indicates the most undesirable, and the highest rung indicates the most desirable state with respect to their standing in the community (community status ladder) and in Hong Kong (Hong Kong ladder)^(17)^. Depressive symptoms were assessed using the Chinese version of the Geriatric Depression Scale (GDS) short form, consisting of fifteen questions relevant to depression including motivation, self-image, losses, agitation and mood. The total GDS score ranges from 0 to 15, and a cut-off of 8 or above was defined as the presence of depressive symptoms^(18,19)^. Cognitive function was assessed using the cognitive part of the Community Screening Instrument for Dementia (CSID)^(20)^. The total CSID score ranges from 0 to 33 and categorises into normal (≥29⋅5), borderline (28⋅4–29⋅49) and probably dementia (<28⋅4). Medical history was obtained based on participants’ self-report of their physician's diagnosis, supplemented by the identification of medications brought to the interviewers. A list of diseases was used to assess the presence of chronic diseases: diabetes, hyperthyroidism, hypothyroidism, osteoporosis, stroke, Parkinson's disease, hypertension, heart attack, angina, congestive heart failure (CHF), chronic obstructive pulmonary disease (COPD), prostatitis, glaucoma, cataracts, gastrointestinal surgery, rheumatoid arthritis (RA), osteoarthritis, gout and cancer. The 12-item Physical Activity Scale for the Elderly (PASE) was used to assess the average number of hours per day spent in leisure, household and occupational physical activities in the last week^(21)^. Activity weights for each item were determined based on the amount of energy spent, and each item score was computed by multiplying the activity weight with daily activity frequency reported. A PASE score was computed by summing each item score, with a higher score indicates a higher physical activity level.

### Dietary patterns

A validated semi-quantitative food frequency questionnaire (FFQ) was used to assess dietary intake^(22)^. The FFQ consisted of 280 food items. A trained interviewer asked each participant to report the frequency and the usual quantity of each food item consumed over the past year. A catalogue of pictures of individual food portions was used to explain portion size to participants. The usual cooking methods of preparing foods (e.g. boiled, stir-fried, steamed and deep-fried) and the type of cooking oil used were asked. The quantity of cooking oil was estimated according to the usual cooking methods of preparing standardised portions of different foods and the usual portion of different foods consumed by the participants.

*A priori* and *a posteriori* dietary pattern scores were calculated, and details of the calculation have been described previously^(23,24)^. The Dietary Quality Index International (DQI-I) was used to assess the quality of diet, as it is an indicator of dietary patterns in relation to health^(25)^ and has been used in a Chinese population^(23)^. The DQI-I score was calculated according to the method described by Kim *et al.*^(25)^. In brief, four major categories, including variety, adequacy, moderation and overall balance, were assessed in the index. In the present study, sufficient information was not available to calculate the category of empty-calorie foods under the category ‘moderation’. Therefore, the total DQI-I score ranges from 0 to 94 instead of 0 to 100, with a higher score indicates better diet quality.

The Dietary Approaches to Stop Hypertension (DASH) diet emphasises intakes of fruits and vegetables, beans, nuts, whole grains and low-fat dairy and limits food high in saturated fat and sugar^(26)^. A DASH score developed by Mellen *et al.* was used to assess adherence to the DASH dietary pattern^(27)^. The score is based on DASH target intakes for nine nutrients including total fat, saturated fat, protein, fibre, cholesterol, calcium, magnesium, potassium and sodium. A score of 1 was given for each nutrient target achieved, and 0⋅5 was given for meeting a nutrient target that was intermediate between the DASH target and the nutrient content of the diet of the control group in the DASH trial. The total DASH score was computed by summing the score for each nutrient target and ranges from 0 to 9. A higher total DASH score indicates better DASH accordance.

The Mediterranean Diet Score (MDS) was calculated using the revised method described by Trichopoulou *et al.* to assess the adherence to the Mediterranean diet^(28)^. Adherence is represented by a scale in which a value of 1 was assigned to the consumption of food groups considered beneficial to health at or above the sex-specific median and below the median for food groups presumed to be detrimental to health. The component of ethanol consumption was scored 1 if daily consumption was between 10 and 50 g for men or 5 and 25 g for women. The total MDS ranges from 0 (minimal adherence to the traditional Mediterranean diet) to 9 (maximal adherence).

Dietary patterns were also derived from our study population using factor analysis. Individual food items from the FFQ were aggregated into thirty-two food groups based on the similarity of type of food and nutrient composition. The food groups were energy adjusted by dividing the energy intake from each food group by the total energy intake and multiplying by 100 and were expressed as percentage contribution to the total energy. The factor scores for each pattern were calculated for each participant by summing the intake of food items weighted by their factor loadings. A higher score indicated greater conformity with the pattern being calculated. Factor analysis identified three dietary patterns in men and women: vegetable–fruit pattern, snack–drink–milk product pattern and meat–fish pattern^(24)^. The ‘vegetable–fruit’ pattern was dominated by frequent intake of vegetables, fruits, legumes, soya and soya products. ‘Snack–drink–milk product’ pattern was characterised by frequent intake of condiments, coffee, fast food, French fries, potato chips, nuts and milk products. ‘Meat–fish’ pattern included frequent intake of dim sum, red and processed meats, poultry, fish and seafood.

### Classification of malnutrition

GLIM criteria, which are composed of three phenotypic and two aetiologic components, were used for the classification of malnutrition^(12)^. At least one phenotypic criterion (non-volitional weight loss, low body mass index (BMI) and reduced muscle mass) and one aetiological criterion (reduced food intake/assimilation and inflammation/disease burden) were required for the classification of malnutrition. In the present study, data of one GLIM phenotypic criterion (non-volitional weight loss) and one aetiologic criterion (reduced food intake/assimilation) were not available from the database. Since it was possible to classify malnutrition as long as the participant met at least one phenotypic criterion and one aetiological criterion, participants were included in the analysis even if some of the data was lacking. Modified GLIM criteria based on two phenotypic components and one aetiologic component were used in the present study. The combination of the phenotypic and aetiologic components for the classification of malnutrition in the present study included: (1) low BMI and inflammation; (2) low BMI and disease burden; (3) reduced muscle mass and inflammation; (4) reduced muscle mass and disease burden.

Body weight was measured with participants wearing light clothing, using the Physician Balance Bean Scale (Healthometer, Illinois, USA). Height was measured using the Holtain Harpenden Standiometer (Holtain Ltd, Crosswell, UK). BMI was calculated as body weight in kilograms divided by height in metre squared. Low BMI was defined using the Asian specific cut-off of <18⋅5 kg/m^2^ if aged <70 years or <20⋅0 kg/m^2^ if ≥70 years^(12)^.

Body composition was measured using the dual-energy X-ray absorptiometry (DXA) (Hologic QDR-4500W, software version 11.2; Hologic, Inc., Waltham, MA, USA). Total appendicular skeletal muscle mass (ASM) was calculated by the sum of lean mass measured in the four limbs, with the operator adjusting the cut lines of the limbs according to specific anatomical landmarks as described by Heymsfield *et al.*^(29)^ Reduced muscle mass was defined as ASM/height^2^: <7⋅0 kg/m^2^ for men and <5⋅4 kg/m^2^ for women^(30)^.

High-sensitivity C-reactive protein (hsCRP) was used as a proxy measure of inflammation^(12)^. Fasting serum samples were collected and stored at −80°C. A commercially available enzyme-linked immunosorbent assay (Vitros Fusion 5.1, Vitros Chemistry Products, USA) was used to measure the hsCRP level in duplicates and was performed by PathLab Co. Ltd. Sex-specific quartiles for hsCRP in our own data were calculated, and the highest quartile was considered as a sex-specific threshold. The presence of inflammation was defined as hsCRP ≥3⋅2 mg/l in men and ≥3⋅8 mg/l in women. Disease burden was defined as the self-reported history of cancer, COPD, CHF and RA, as these conditions are associated with chronic or recurrent inflammation of a mild-to-moderate degree^(12)^. The aetiological criterion was met if the participants with either the presence of inflammation or disease burden.

### Statistical analysis

Characteristics of participants are described as mean and standard deviation (sd) for continuous variables and as numbers and percentages (%) for categorical variables. Non-normally distributed variables are presented as the median and interquartile range (IQR). Independent Student's *t*-test and *χ*^2^ test were used to examine the baseline differences in characteristics between participants included and participants excluded for data analysis. The differences in characteristics and dietary patterns between malnourished and non-malnourished participants were examined using the *χ*^2^ test for categorical variables and by t-test for continuous variables.

The association between each dietary pattern and the presence of malnutrition was analysed using binary logistic regression models. The DQI-I score and the three dietary pattern scores derived by the factor analysis were first stratified into tertiles based on the distribution of each sex. The DASH score was divided into two levels of adherence, using at least 4⋅5 as a cut-off value^(27)^. The MDS score was divided into three levels of adherence, namely low (0–3), medium (4–5) and high (6–9)^(28,31)^. Test for trend was examined by entering tertiles of each dietary pattern or the DASH adherence or three levels of MDS as a continuous variable in all models. We also examined the interaction between sex and each dietary pattern by the addition of cross-product terms to the regression models. The interaction was significant, and all subsequent analyses were stratified by sex. Model 1 was the crude model. Age, daily energy intake and independent variables with *P* < 0⋅1 in the univariate analyses were considered to be potential confounders for adjustment and entered into the multiple logistic regression model. For men, Model 2 was adjusted for age (continuous), BMI (continuous), current smoker (yes *v.* no), current drinker (yes *v.* no), marital status (married/cohabited *v.* widowed/separated/divorced/single/never married), subjective social status community status ladder (continuous), number of chronic diseases (0–1 *v.* ≥2), depressive symptoms (yes *v.* no), CSID category (normal *v.* borderline *v.* probably dementia), PASE score (continuous) and daily energy intake (continuous). For women, Model 2 was adjusted for age (continuous), BMI (continuous), education level (primary or below *v.* secondary and above), subjective social status community status ladder (continuous), number of chronic diseases (0–1 *v.* ≥2), CSID category (normal *v.* borderline *v.* probably dementia), PASE score (continuous) and daily energy intake (continuous). Data are presented as odds ratio (OR) and 95 % confidence intervals (CI).

Statistical analyses were performed using the statistical package SPSS version 24.0 (IBM SPSS Statistics for Windows, Version 24.0. Armonk, NY, USA: IBM Corp.). All tests were two-sided. To account for multiple testing for various dietary patterns, findings were considered statistically significant if they passed the Bonferroni cut-off of *P* < 0⋅008 (*α* 0⋅05/6).

## Results

Excluded participants were older, had lower BMI, had higher educational attainment and had lower subjective social status (community ladder) in comparison to included participants (*P* < 0⋅05). Excluded participants were also more likely to be men and to be current smokers than those who were included in the analysis (*P* < 0⋅05) (details not shown).

Among 3694 participants, 293 men (16⋅2 %) and 104 women (5⋅5 %) were classified as malnutrition according to the GLIM criteria. The prevalence of each phenotypic component and aetiologic component and their combinations for the classification of malnutrition are presented in Supplementary Table S1 of Supplementary material. The characteristics of participants with malnutrition and participants without malnutrition by sex are shown in Table 1. Men with malnutrition were more likely to be older, have lower BMI, being a current smoker, have lower subjective social status, ≥2 chronic diseases, depressive symptoms, probable dementia, lower PASE score and less likely to be married and being physically active than men without malnutrition. Women with malnutrition were more likely to be older, have lower BMI, ≥2 chronic diseases, lower PASE score and less likely to have probable dementia than women without malnutrition.

**Table 1.** Characteristics of 3694 community-dwelling older adults by malnutrition status

	All					Men					Women				
	Non-malnourished (*n* 3297)		Malnourished (*n* 397)		P*	Non-malnourished (*n* 1517)		Malnourished (*n* 293)		P*	Non-malnourished (*n* 1780)		Malnourished (*n* 104)		P*
	Median/mean	IQR/SD	Median/mean	IQR/SD		Median/mean	IQR/SD	Median/mean	IQR/SD		Median/mean	IQR/SD	Median/mean	IQR/SD	
Age, year	71	68–76	73	70–78	**<0⋅001**	71	68–75	73	70–78	**<0⋅001**	71	68–76	74	69–78	**0⋅017**
Age group, %					**<0⋅001**					**<0⋅001**					**0⋅042**
65–69 years	34⋅8		23⋅9			35⋅4		23⋅5			34⋅4		25⋅0		
70–74 years	34⋅6		32⋅7			35⋅9		33⋅1			33⋅5		31⋅7		
≥75 years	30⋅5		43⋅3			28⋅7		43⋅3			32⋅1		43⋅3		
BMI, kg/m^2^	24⋅3	3⋅1	20⋅9	2⋅5	**<0⋅001**	24⋅2	2⋅9	21⋅2	2⋅4	**<0⋅001**	24⋅4	3⋅3	20⋅2	2⋅7	**<0⋅001**
Current smoker, %	5⋅9		12⋅1		**<0⋅001**	10⋅7		15⋅0		**0⋅032**	1⋅7		3⋅8		0⋅122
Current drinker, %	12⋅7		14⋅9		0⋅221	24⋅4		19⋅5		0⋅068	2⋅7		1⋅9		1⋅000
Live alone, %	11⋅0		9⋅3		0⋅298	4⋅4		5⋅8		0⋅277	16⋅7		19⋅2		0⋅510
Being married, %	69⋅9		74⋅3		0⋅070	88⋅8		83⋅6		**0⋅013**	53⋅8		48⋅1		0⋅254
Education level, %					**0⋅024**					0⋅267					0⋅095
Primary or below	72⋅9		67⋅5			60⋅7		64⋅2			83⋅3		76⋅9		
Secondary and above	27⋅1		32⋅5			39⋅3		35⋅8			16⋅7		23⋅1		
Subjective social status, rung															
Community Ladder	6⋅9	2⋅2	6⋅3	2⋅3	**<0⋅001**	6⋅4	2⋅2	6⋅1	2⋅3	**0⋅017**	7⋅4	2⋅1	7⋅0	2⋅1	0⋅077
Hong Kong Ladder	4⋅6	1⋅9	4⋅4	2⋅0	0⋅077	4⋅5	1⋅9	4⋅3	2⋅0	0⋅311	4⋅6	1⋅9	4⋅5	2⋅1	0⋅400
No. of chronic diseases^a^, %					**<0⋅001**					**<0⋅001**					**0⋅003**
0–1	44⋅8		31⋅0			45⋅1		31⋅4			44⋅5		29⋅8		
≥2	55⋅2		69⋅0			54⋅9		68⋅6			55⋅5		70⋅2		
Depressive symptoms, %	9⋅0		12⋅1		**0⋅044**	7⋅7		11⋅6		**0⋅025**	10⋅1		13⋅5		0⋅276
CSID category, %					**0⋅003**					**0⋅031**					**0⋅030**
Normal	72⋅3		80⋅1			88⋅7		83⋅3			58⋅3		71⋅2		
Borderline	11⋅9		9⋅6			6⋅7		9⋅6			16⋅3		9⋅6		
Probable dementia	15⋅8		10⋅3			4⋅5		7⋅2			25⋅4		19⋅2		
PASE score	85	63–111	79	60–108	**0⋅003**	93	61–126	81	58–115	**0⋅002**	85	64–103	76	59–94	**0⋅008**
Daily energy intake, kcal	1827	593	1934	549	**0⋅001**	2112	600	2054	543	0⋅122	1584	465	1595	407	0⋅810

BMI, body mass index; CSID, Cognitive Screening Instrument for Dementia; PASE, Physical Activity Scale of the Elderly; SD, standard deviation.

a Including diabetes, hyperthyroidism, hypothyroidism, osteoporosis, stroke, Parkinson's disease, hypertension, heart attack, angina, congestive heart failure, chronic obstructive pulmonary disease, prostatitis, glaucoma, cataracts, gastrointestinal surgery, rheumatoid arthritis, osteoarthritis, gout and cancer.

* *P*-value between non-malnourished participants *v.* malnourished participants. Values are presented as percentages, median and interquartile range, or mean and standard deviation.

Table 2 shows the dietary patterns of participants with and without malnutrition by sex. Men with malnutrition had lower DQI-I score, and DASH score compared with men without malnutrition. After the Bonferroni correction, only the DQI-I score was statistically significantly different between men with and without malnutrition. None of the dietary pattern scores differed among women with and without malnutrition.

**Table 2.** Dietary patterns by malnutrition status and sex among 3694 community-dwelling older adults

	All					Men					Women				
Dietary patterns	Non-malnourished (*n* 3297)		Malnourished (*n* 397)		P*	Non-malnourished (*n* 1517)		Malnourished (*n* 293)		P*	Non-malnourished (*n* 1780)		Malnourished (*n* 104)		P*
	Mean	SD	Mean	SD		Mean	SD	Mean	SD		Mean	SD	Mean	SD	
DQI-I score (0–94)	64⋅6	9⋅5	62⋅4	10⋅1	**<0⋅001**	64⋅1	9⋅7	61⋅8	10⋅1	**<0⋅001**	65⋅0	9⋅3	64⋅0	10⋅1	0⋅272
DASH score (0–9)	4⋅0	1⋅3	3⋅7	1⋅2	**<0⋅001**	3⋅7	1⋅2	3⋅5	1⋅2	0⋅020^†^	4⋅3	1⋅3	4⋅1	1⋅4	0⋅056
MDS score (0–9)	4⋅1	1⋅5	4⋅0	1⋅5	0⋅195	4⋅2	1⋅5	4⋅0	1⋅5	0⋅165	4⋅1	1⋅5	4⋅0	1⋅6	0⋅475
Vegetable–fruit	0⋅01	0⋅97	−0⋅11	1⋅12	0⋅032^†^	−0⋅19	0⋅91	−0⋅28	1⋅00	0⋅164	0⋅19	0⋅97	0⋅35	1⋅30	0⋅116
Snack–drink– milk product	−0⋅01	1⋅01	0⋅00	0⋅97	0⋅932	0⋅19	1⋅02	0⋅09	1⋅02	0⋅108	−0⋅17	0⋅96	−0⋅25	0⋅77	0⋅426
Meat–fish	−0⋅02	1⋅00	0⋅14	1⋅04	**0⋅003**	0⋅17	1⋅04	0⋅26	1⋅06	0⋅173	−0⋅18	0⋅93	−0⋅20	0⋅87	0⋅786

DQI-I, Diet Quality Index International; DASH, Dietary Approaches to Stop Hypertension; MDS, The Mediterranean Diet Score.

* Independent *t*-test or Mann–Whitney *U* test as appropriate. *P*<0⋅008 were considered statistically significant after the Bonferroni correction and indicated in bold.

† *P* < 0⋅05.

Table 3 shows the logistic regression results of each dietary pattern and malnutrition. Men in the highest tertile of DQI-I score (adjusted OR 0⋅59, 95 % CI 0⋅41, 0⋅86, *P*-trend 0⋅005) and ‘vegetable–fruit’ dietary pattern score (adjusted OR 0⋅70, 95 % CI 0⋅50, 0⋅99, *P*-trend 0⋅035) had reduced risk of malnutrition, compared with men in the lowest tertile. Men in the highest tertile of ‘meat–fish’ dietary pattern score showed an increased risk of malnutrition compared with men in the lowest tertile (adjusted OR 1⋅51, 95 % CI 1⋅06, 2⋅15, *P*-trend 0⋅023). After the Bonferroni correction, only the DQI-I score remained statistically significant in its association with malnutrition (*P*-trend < 0⋅008). In women, higher adherence to the DASH diet was associated with a lower risk of malnutrition (adjusted OR 0⋅61, 95 % CI 0⋅38, 0⋅97, *P* 0⋅038). After the Bonferroni correction, this association was no longer statistically significant. The associations for the DQI-I score and ‘meat–fish’ dietary pattern and malnutrition were in the same direction as in men. In contrast, women in the highest tertile of ‘vegetable–fruit’ dietary pattern score had an increased risk of malnutrition compared with women in the lowest tertile. However, these associations did not reach statistical significance in women either with or without the Bonferroni correction. A higher MDS score and a lower ‘snack–drink–milk product’ dietary pattern score appeared to be associated with a higher risk of malnutrition in both genders, although the associations were not statistically significant with or without the Bonferroni correction.

**Table 3.** Association between dietary patterns and malnutrition in 3694 community-dwelling older adults

	Men						Women					
Dietary patterns	Model 1			Model 2			Model 1			Model 2		
	OR	95% CI	*P*-trend	OR	95% CI	*P*-trend	OR	95% CI	*P*-trend	OR	95% CI	*P*-trend
DQI-I score			**0**⋅**001**			**0⋅005**			0⋅214			0⋅215
T1	Ref.	Ref.		Ref.	Ref.		Ref.	Ref.		Ref.	Ref.	
T2	0⋅90	0⋅67–1⋅22		0⋅93	0⋅65–1⋅32		0⋅68	0⋅42–1⋅10		0⋅67	0⋅38–1⋅18	
T3	**0**⋅**60**	**0⋅44–0⋅82**		**0**⋅**59**	**0⋅41–0⋅86**		0⋅75	0⋅47–1⋅20		0⋅71	0⋅40–1⋅24	
DASH score			0⋅111			0⋅057			0⋅050			0⋅038*
Low (≤4⋅0)	Ref.	Ref.		Ref.	Ref.		Ref.	Ref.		Ref.	Ref.	
High (≥4⋅5)	0⋅79	0⋅59–1⋅06		0⋅73	0⋅53–1⋅01		0⋅67	0⋅45–1⋅00		0⋅61	0⋅38–0⋅97	
MDS			0⋅271			0⋅787			0⋅713			0⋅866
0–3	Ref.	Ref.		Ref.	Ref.		Ref.	Ref.		Ref.	Ref.	
4–5	0⋅98	0⋅74–1⋅29		1⋅07	0⋅77–1⋅48		0⋅83	0⋅54–1⋅29		0⋅84	0⋅50–1⋅41	
6–9	0⋅79	0⋅55–1⋅15		1⋅05	0⋅67–1⋅62		0⋅95	0⋅55–1⋅65		1⋅14	0⋅59–2⋅19	
Vegetable-fruit			0⋅013*			0⋅035*			0⋅323			0⋅378
T1	Ref.	Ref.		Ref.	Ref.		Ref.	Ref.		Ref.	Ref.	
T2	**0**⋅**56**	**0⋅41–0⋅77**		**0**⋅**50**	**0⋅35–0⋅72**		1⋅00	0⋅61–1⋅65		0⋅96	0⋅54–1⋅73	
T3	0⋅70	0⋅52–0⋅94*		0⋅70	0⋅50–0⋅99*		1⋅27	0⋅79–2⋅05		1⋅28	0⋅73–2⋅26	
Snack-drink- milk-product			0⋅101			0⋅166			0⋅711			0⋅162
T1	Ref.	Ref.		Ref.	Ref.		Ref.	Ref.		Ref.	Ref.	
T2	1⋅02	0⋅76–1⋅38		0⋅93	0⋅66–1⋅31		1⋅06	0⋅66–1⋅71		0⋅84	0⋅48–1⋅48	
T3	0⋅77	0⋅56–1⋅05		0⋅77	0⋅53–1⋅11		0⋅91	0⋅56–1⋅49		0⋅64	0⋅34–1⋅20	
Meat-fish			0⋅138			0⋅023*			0⋅902			0⋅297
T1	Ref.	Ref.		Ref.	Ref.		Ref.	Ref.		Ref.	Ref.	
T2	1⋅05	0⋅77–1⋅44		1⋅07	0⋅75–1⋅54		0⋅85	0⋅52–1⋅40		1⋅01	0⋅57–1⋅79	
T3	1⋅26	0⋅93–1⋅71		1⋅51	1⋅06–2⋅15*		1⋅03	0⋅64–1⋅65		1⋅35	0⋅77–2⋅36	

CI, confidence interval; DQI-I, Diet Quality Index International; DASH, Dietary Approaches to Stop Hypertension; MDS, The Mediterranean Diet Score; OR, odds ratio; T1, first tertile; T2, second tertile; T3, third tertile.

Model 1: crude model; Model 2 (men): adjusted for age, BMI, current smoker (yes/no), current drinker (yes/no), being married (yes/no), subjective social status (community ladder), CSID category (normal/borderline/probable dementia), number of chronic diseases (0–1/ ≥2), depressive symptoms (yes/no), PASE score and daily energy intake; Model 2 (women): adjusted for age, BMI, education level (primary or below/secondary and above), subjective social status (community ladder), CSID category (normal/borderline/probable dementia), number of chronic diseases (0–1/ ≥2), PASE score and daily energy intake. *P*<0⋅008 were considered statistically significant after the Bonferroni correction and indicated in bold.

*P<0⋅05.

## Discussion

Our findings supported that healthier dietary patterns were associated with malnutrition according to the GLIM criteria in Chinese community-dwelling older adults. In men, a higher DQI-I score, a higher ‘vegetable–fruit’ dietary pattern score and a lower ‘meat–fish’ dietary pattern score were associated with a lower risk of malnutrition. In women, higher adherence to the DASH diet was associated with a lower risk of malnutrition. However, after the Bonferroni correction, the associations remained statistically significant only in men for the DQI-I score.

The gender difference in the results may be explained by several factors. Men, in general, showed less healthy dietary patterns and lifestyles than women. Therefore, data from men may be more heterogeneous compared with women, in which significant associations between dietary patterns and malnutrition were more easily detected in the present study. Another factor may be due to the fewer number of women being classified as malnutrition compared with men. These results may reflect the more unfavourable eating habits of older men (particularly those who lived alone) of not cooking at home but relying on fast food and takeaway lunch boxes^(32–34)^. Taking away is an alternative for people who do not like cooking nor eating alone in restaurants^(35)^. Further exploration of our data showed that men who lived alone had lower dietary quality as assessed by the ‘vegetable–fruit’ dietary pattern score than those who lived with others. Although the dietary data were collected almost 20 years ago, research has shown that the dietary habits of older adults are relatively stable over time^(36–38)^.

To the best of our knowledge, the present study was the first study to examine different dietary patterns on malnutrition according to the GLIM criteria in community-dwelling older adults. Direct comparison with similar studies was therefore not feasible due to the different criteria used to classify malnutrition such as MNA^(9,39,40)^, low BMI and/or involuntary weight loss^(8)^. In general, previous studies supported our findings that higher dietary variety^(39)^ and better diet quality^(10,11)^ were associated with a lower risk of malnutrition.

As our participants that were identified as malnourished fulfilled the aetiologic criterion of inflammation or disease burden; therefore, the influence of diet on disease-related malnutrition according to the GLIM criteria is expected. The literature has shown the association between diet and inflammatory statuses such as high hsCRP and disease burden^(15,41,42)^. In Chinese community-dwelling older adults, a higher DQI-I score was associated with a lower hsCRP level in men but this association was not observed in women^(15)^. A higher meat and instant food dietary pattern score has also been reported to be associated with higher hsCRP levels in Taiwanese community-dwelling older adults^(43)^. Furthermore, a systematic review supports the notion that a positive association exists between meat-based diets and low-grade chronic inflammation^(44)^, which may contribute to the higher levels of overall inflammation and oxidative stress that are often associated with muscle ageing and chronic diseases^(45,46)^. Although the positive association between the ‘meat–fish’ pattern and malnutrition was not significant after the Bonferroni correction, the direction of the association was somehow reasonable. Inflammation contributes to malnutrition through associated anorexia and decreased food intake, as well as altered metabolism with an elevation of resting energy expenditure and increased muscle catabolism^(12)^. The ‘meat–fish’ pattern had high factor loadings for dim sum, red and processed meats, poultry, fish and seafood; however, it did not load high on vegetables and fruits. Although adequate protein intake may play a role in counteracting disease-related malnutrition, too much protein intake may raise an acidic environment. It has been proposed that metabolic acidosis can cause tissue damage, which can further initiate inflammatory responses^(47)^. Vegetables and fruits could provide antioxidants to quench free radicals and reduce damage from reactive oxygen species and inflammatory status^(48)^. The absence of these foods may partly explain the positive associations observed with the ‘meat–fish’ pattern. The findings suggest that the balance of total diet and variety may be most important in determining its role on the risk of disease-related malnutrition.

It should be noted that our participants that were identified as malnourished fulfilled more often the phenotypic criterion of low muscle mass than of low BMI. A previous study showed that adherence to a Western dietary pattern (high in red meat, fried foods and high-fat dairy) was associated with higher D_3_Cr muscle mass, while the healthy dietary pattern (high in fruits, vegetables, whole grains and lean meats) was not associated with D_3_Cr muscle mass or appendicular muscle mass assessed using DXA in older men^(49)^. The findings are somewhat different from those found in the present study, in which a lower adherence to ‘meat–fish’ dietary pattern was associated with a lower risk of malnutrition. The previous study did not specify the aetiology of low muscle mass. Therefore, the different findings may be explained by inflammation/disease burden as the aetiology in our participants as described earlier, compared with other aetiologies of low muscle mass such as reduced food intake or assimilation^(12)^. However, the absence of associations between DASH, MDS and malnutrition in the present study were similar to those reported by Huang *et al.* in which DASH and MDS were not associated with muscle mass among Japanese older adults^(50)^. The MDS uses the group median intake of each component as the cut-off value for calculation. This scoring system may result in bias in that the MDS calculation is based on cohort- and sex-specific median values across food categories of the studied population; therefore, the score may not be related to a healthy level of intake *per se*^(51)^.

Given the association between DQI-I score and malnutrition found in the present study, it appears that improving dietary quality could potentially reduce the risk of malnutrition in older adults, particularly in the aspects of a high variety of foods, adequate intake of healthy foods, moderate intake of nutrients that are related to chronic diseases and an overall balance in macronutrients and fatty acids^(25)^. Nonetheless, it should be noted that older adults often encounter barriers to healthy eating, including social determinants (e.g. eating alone), health problems (e.g. impaired mobility, vision and dental problems) and decreased financial and social support. This age group also seems to struggle with change more than other age groups, as ‘resistance to change’ and ‘giving up liked foods’ have been identified as barriers unique to this age group^(52)^. This emphasises the importance of promoting healthy eating through a life-course approach and the need for healthcare professionals to address these age-specific barriers during health promotion^(53)^.

The strengths of the present study included the analysis of various dietary patterns, which allowed assessment of the interaction among synergistic components in the diet and facilitated the development of food-based dietary guidelines^(7,54)^. A wide range of participants’ lifestyle factors allowed us to adjust for their confounding effects in the analyses. However, the cross-sectional data cannot infer a causal relationship because data of dietary patterns and malnutrition according to the GLIM criteria were obtained at the same time. Second, dietary data were collected using FFQ which may be subject to recall bias. Third, our participants were of higher educational level and were able to travel to the study site; therefore, they were more likely to be more health-conscious and have a better diet compared to the general population. Therefore, the results may not be extrapolated to the general target population or applicable to other countries with different cultures and dietary cultures. Fourth, although a statistically significant difference of DQI-I score was found between men with and without malnutrition, whether it is a clinically relevant difference is not well-defined in the literature. Lastly, data of weight loss and reduced food intake were not available in our dataset. These criteria are important, as it has been shown that Chinese community-dwelling older adults who had a lack of appetite and meal skipping behavior (may imply insufficient food intake and subsequent weight loss) had a higher risk of malnutrition defined by the MNA^(55)^. However, based on our data collected at the 2-year follow-up, the prevalence of weight loss (10⋅2 %) and reduced food intake (6⋅0 %) was low. Therefore, although the prevalence of malnutrition may have been underestimated using the modified GLIM criteria, we do not expect a huge difference in the association between dietary patterns and malnutrition if the original GLIM criteria was applied in our population.

## Conclusions

In community-dwelling Chinese older adults in Hong Kong, a better diet quality as characterised by a higher DQI-I score was associated with a lower risk of malnutrition according to the modified GLIM criteria in men, whereas dietary patterns were not associated with malnutrition in women. Nutritional strategies for the prevention and management of disease-related malnutrition could potentially be targeted on dietary quality in the aspects of variety, adequacy, moderation and overall balance, with the consideration of age-specific barriers to healthy eating.

## Supplementary material

For supplementary material accompanying this paper visit http://dx.doi.org/10.1017/jns.2021.64. click here to view supplementary material
