# Supplementary material for: Certain dietary patterns are associated with GLIM criteria among Chinese community-dwelling older adults: a cross-sectional analysis
Source: J Nutr Sci. 2021 Aug 27;10:e69. doi: 10.1017/jns.2021.64 (PMC8411258; doi:10.1017/jns.2021.64)
Supplement: Supplementary file 1 [file S2048679021000641sup001.docx]

**Supplementary Table 1.** Prevalence of each phenotypic component and etiologic component and their combinations for the classification of malnutrition

| Criteria | N | Prevalence, n (%) |
| --- | --- | --- |
| **Phenotypic component** |  |  |
| Low BMI | 3694 | 323 (8.7) |
| Reduced muscle mass | 3694 | 890 (24.1) |
| Low BMI or Reduced muscle mass | 3694 | 955 (25.9) |
| **Etiologic component** |  |  |
| Disease burden^*^ | 3694 | 671 (18.2) |
| Inflammation† | 2811 | 710 (25.3) |
| Disease burdena or Inflammation† | 2999 | 1229 (41.0) |
| **GLIM criteria** |  |  |
| 1. Low BMI & Inflammation | 3669 | 54 (1.5) |
| 1. Low BMI & Disease burden | 3694 | 90 (2.4) |
| 1. Reduced muscle mass & Inflammation | 3631 | 203 (5.6) |
| 1. Reduced muscle mass & Disease burden | 3694 | 238 (6.4) |
| **Any meeting of the GLIM criteria** | **3694** | **397 (10.7)** |

BMI, body mass index; GLIM, Global Leadership Initiative on Malnutrition
^*^self-reported history of cancer, chronic obstructive pulmonary disease, congestive heart failure and/or rheumatoid arthritis; †assessed by hsCRP≥ sex-specific highest quartile (≥3.2 mg/L for men and ≥3.8 mg/L for women)
